# Ultrafast fiber laser at 1570 nm based on organic material as saturable absorber

**DOI:** 10.1038/s41598-022-17724-9

**Published:** 2022-08-02

**Authors:** Ahmed Shakir Al-Hiti, Zian Cheak Tiu, M. Yasin, S. W. Harun

**Affiliations:** 1 Department of Electrical Engineering, Faculty of Engineering, University of Malaya, 50630 Kuala Lumpur, Malaysia; 2 Faculty of Engineering and Quantity Surveying, INTI International University, 71800 Nilai, Negeri Sembilan Malaysia; 3 Department of Physics, Faculty of Science and Technology, Airlangga University, Surabaya, 60115 Indonesia

**Keywords:** Engineering, Optics and photonics

## Abstract

In this work, we demonstrated Poly(3,4-ethylenedioxythiophene): poly(styrenesulfonate) (PEDOT: PSS) as a saturable absorber (SA) to produce mode-locking operation in different length of Erbium-doped fiber laser (EDFL). The PEDOT: PSS was embedded into polyvinyl alcohol to form a thin film that acts as an absorber into the laser setup. The three different mode-locked EDFL were successfully demonstrated with different cavity length and output coupler ratio. The pulse repetition rate/width of 3.417 MHz/710 fs, 4.831 MHz/510 fs, and 6.049 MHz/460 fs were obtained by utilizing optical coupler/ cavity length of 20:80/60.7 m, 10:90/42.7 m, and 5:95/33.7 m, respectively. All experiments generated a stable and mode-locked operation at a central wavelength of 1570.76 nm, 1570.3 nm, and 1569.95 nm with 3 dB bandwidth of 4.8 nm, 5.6 nm, and 6.5 nm, respectively. The long-time stability of the ultrafast fiber lasers was investigated for each setup via 120 min. The proposed PEDOT: PSS has proven as a promising material to induce mode-locking operation in different fiber laser setup.

## Introduction

A variety of photonics systems such as nonlinear optics and all-fiber seeing have been revolutionized with distinctive characteristics of erbium-doped fiber lasers (EDFLs). The tremendous growth of research interests in EDFLs are attributed to their ability to produce tunable outputs with perfect beam quality, low insertion loss, high output power, and narrow linewidth^1,2^. These fiber lasers can be operated in either pulse mode or continuous wave (CW). Pulsed EDFLs are referring to ultrafast lasers with high peak power, operate in Q-switching^3^ or mode-locking^4^ operation. The mode-locked EDFLs have been widely used in high-capacity optical communication applications due to their distinctive ability to produce femtosecond pulse through active or passive techniques^5^. Active technique required an external modulators and electronic components, such as photoelectric modulators and acoustic optics^6^, which make the system inflexible and expensive. While passive technique provides a more airtight and diversified solution. Saturable absorbers (SAs) are a key to generate ultrafast laser in the passive technique that can classified into real and artificial SAs. Artificial SAs are formation of optical components, such as nonlinear polarization evolution (NPE)^7^, nonlinear amplification loop mirrors (NALMs)^8^, and nonlinear optical loop mirrors (NOLMs)^9^. Artificial SAs required the formation of multiple optical components and sensitivity to environmental perturbation, which constrained its feasibility. Semiconductors saturable absorbers mirrors (SESAMs)^10^ have been used as real SAs. Unfortunately, SESAMs suffer from many drawbacks, including high cost, narrow operating bandwidth, low-damage threshold, and complex setup^11^. Therefore, emerging material SAs becoming the main research focus to induce ultrafast phenomenon in fiber laser system. There are many two-dimensional (2D) and emerging materials have been proposed as SAs to generate pulsed laser, which including graphene^12^, carbon nanotubes (CNTs)^13^, black phosphorus (BP)^14^, transition metal dichalcogenides (TMDs)^15–17^, and topological insulators (TIs)^18–20^. These materials have proven a great potential as SA with their exceptional performance in absorption^21^, size^22^, chemical stability^23^, and recovery time^24^. Recently, Organic Materials (OMs) have been highlighted as new emerging material, which exhibit great flexibility, thermal stability, and film-forming ability. These properties allow the use of OMs in forefront technologies. Unsurprisingly, the OM application is extending to ultrafast laser applications. For instance, polymer of poly (3,4-ethylenedioxythiophene) polystyrene sulfonate (PEDOT: PSS), a member of OM, have been reported to induce picosecond pulse in fiber laser system^25^. However, the investigation of OM’s potential to induce ultrafast laser is still handful as compared to other emerging materials.

In this experiment, we demonstrated a PEDOT: PSS-based SA as a passive modulator to generate mode-locking operation in EDFL L-band region. The PEDOT: PSS SA was fabricated by embedding PEDOT: PSS powder within a Polyvinyl alcohol (PVA) host polymer. Owing to the physical flexibility, film-forming characteristic, and thermal stability, PEDOT: PSS is showing a great potential in versatile applications^26,27^, particularly in photonic applications. The proposed SA film achieved excellent results with a modulation depth of 50% and a saturation intensity of 32 MW/cm^2^. Stable mode-locked in different EDFL setup were obtained with a pulse width/maximum output power of 710 fs/20.07 mW, 510 fs/15.82 mW, and 460 fs/11.89 mW by utilizing an optical coupler (OC)/ cavity length of 20:80/60.7 m, 10:90/42.7 m, and 5:95/33.7 m, respectively.

## Sample preparation and characterization

**Figure 1:**
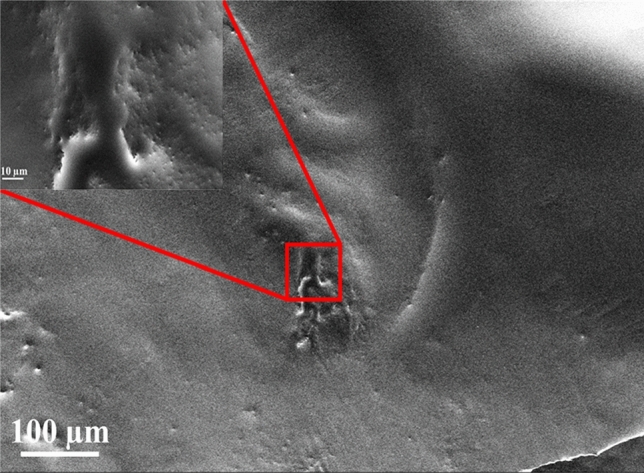
The SEM image of the absorber film.

PEDOT is one of the most explored and widely utilized OM due to its stability in the air, resistance to moisture, and high conductivity. It also can be polymerized from 3,4-ethylenedioxythiophene (EDOT) electrochemically or chemically. However, PEDOT is doped with counter ions of small molecules that are insoluble in any solvent and elusive in large scale fabrication ^28^. When polymerization is executed with aquatic polyelectrolyte poly(styrenesulfonate) (PSS), it will be water-dispersible with good film formatting characteristics, stable, and is easy to fabricate. The PSS acts as a mould by counter ion of charge balancing and polymerization which maintain dispersed of cationic PEDOT segments in an aqueous medium. The hydrophilic PSS and hydrophobic PEDOT nature led to the core–shell structure ^29,30^. The molecular weight of PSS and PEDOT is about 400,000 g/mol and 1000–2500 g/mol, respectively. The PVA is a polymer material that has unique and excellent characteristics such as biodegradable, non-poisonous, and fully being water-soluble^31^. The fabrication procedure of absorber film was by dissolving 1 mg of PEDOT: PSS into 10 ml of deionized (DI) water at 60 °C for 60 min. During the fabrication process of the PEDOT: PSS solution, acetone was added to dissolve the PEDOT: PSS nano-powder. Next, we prepared the PVA by mixing 1 g of PVA in 100 ml of DI water with stirring in an ultrasonic agitator for about 120 min. Then, a solution of the PEDOT: PSS PVA mixture was prepared over 5 ml of the PVA solution after fabrication process into PEDOT: PSS solution by stirring at 45 °C for ~ 180 min. Finally, the solution mixture was decanted into a plastic mould with 60 mm diameter and dried for 3 days to form a thin film. The film thickness was measured to be around 50 μm. In this work, PEDOT: PSS was prepared in different stirring times and with different weight ratios. The best performance of a pulsed laser was obtained by this process described above in a controlled laboratory environment. Figure 1 shows two different samples of SEM images were taken for the proposed SA film. The first sample showed that the PEDOT: PSS-particles were distributed homogeneously with PVA within a range of 100 µm. Small particles were discovered which belong to PEDOT: PSS agglomeration powder. The inset is SEM image taken at a higher magnification of the SA film in the 10 μm range, showing many creases on the surface which resulted from annealing procedure at room temperature. The thickness of the SA film is measured to be around 50 µm. It has an insertion loss of about 0.5 dB with negligible polarization dependent loss (PDL). We used the same SA in the three different experiments.

**Figure 2:**
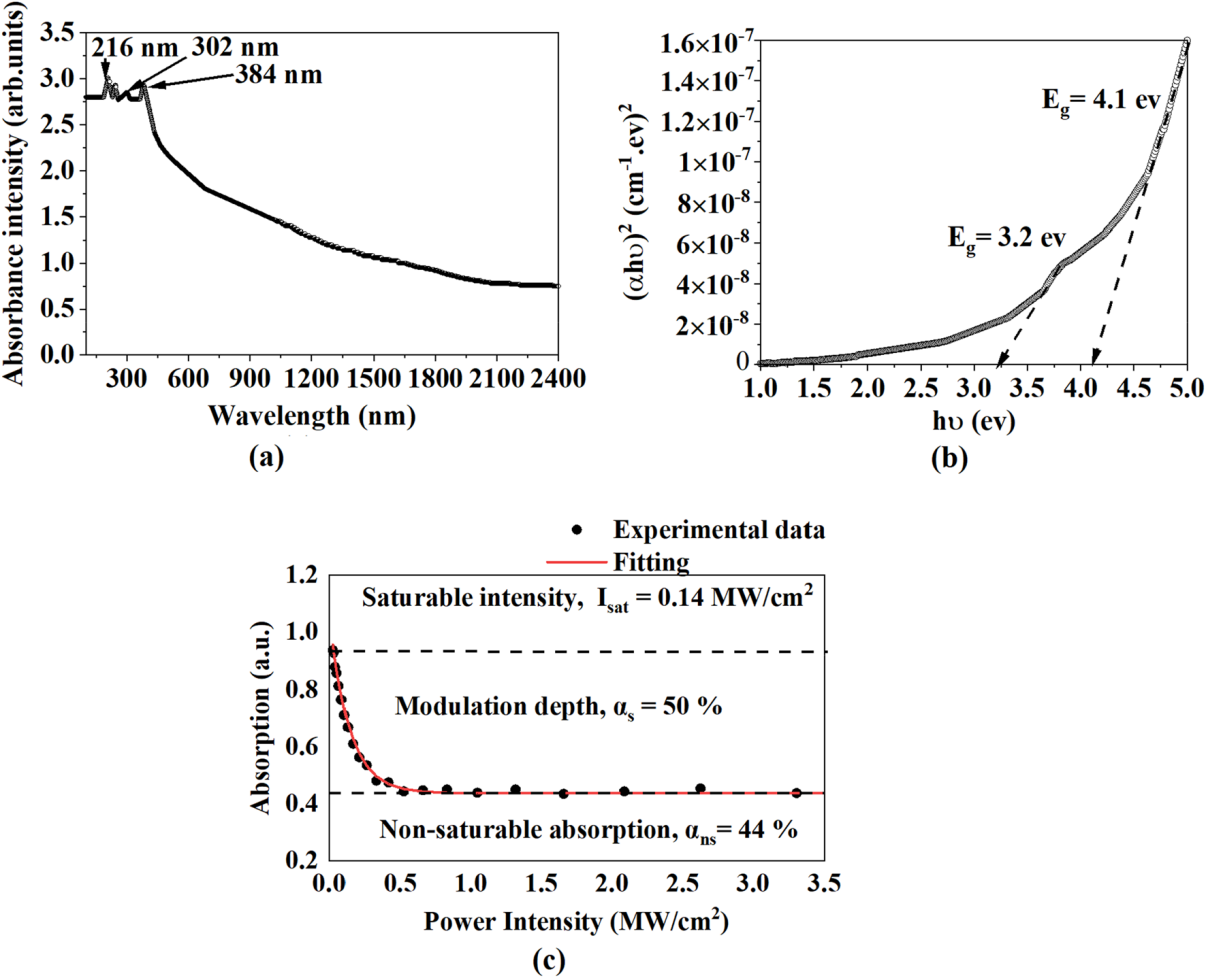
The characterization of SA film (**a**) The optical absorption spectrum (**b**) The optical band gap curve, and (**c**) The nonlinear absorption curve.

Figure 2a illustrations the optical absorbance spectrum of SA film. Three broad and asymmetric peaks centered at 216 nm, 302 nm, and 384 nm, respectively. These peaks correspond to $\pi \to {\pi }^{*}$ transition of PSS, PVA, and PEDOT molecules due to unsaturated bonds^32,33^. The optical bandgap (E_g_) of the SA film can be calculated based on the equation (*αhv)*^*2*^ = *B(hv-E*_*g*_), as *n* is the equal 2 for direct transition, *α* is absorption coefficient, *B* is constant relative, and *hv* is the photon energy which can be measured via following equation *α(v)* = *2.303* × *Abs (λ)/d,* as* d* is the SA film thickness. We can obtain the optical bandgap by linear extrapolation at the axis of (*αhv)*^*2*^ versus *hv*. Figure 2b shows two bandgap values that were gained at 3.2 and 4.1 eV and belonged to the modified PVA and PEDOT: PSS^34,35^.

The nonlinear absorption of the PEDOT: PSS PVA was investigated utilizing a standard 2-arm transmission measurement method. A stable mode-locked laser was utilized as a pulsed source operating at 1570 nm. The pulse repetition rate and pulse duration of the laser were 6.049 MHz and 460 fs respectively. The pulse source was amplified by EDFA and connected to an attenuator to alter the laser output power. Then, the 3 dB coupler was used to split the output power. One port was utilized for reference and another port was utilized for power-dependent transmission measurement of the SA film. The modulation depth of SA film was achieved about 50% with a saturation intensity of 0.14 MW/cm^2^ which is higher than other recent work^36–38^ as shown in Fig. 2c.

## Laser configuration

**Figure 3:**
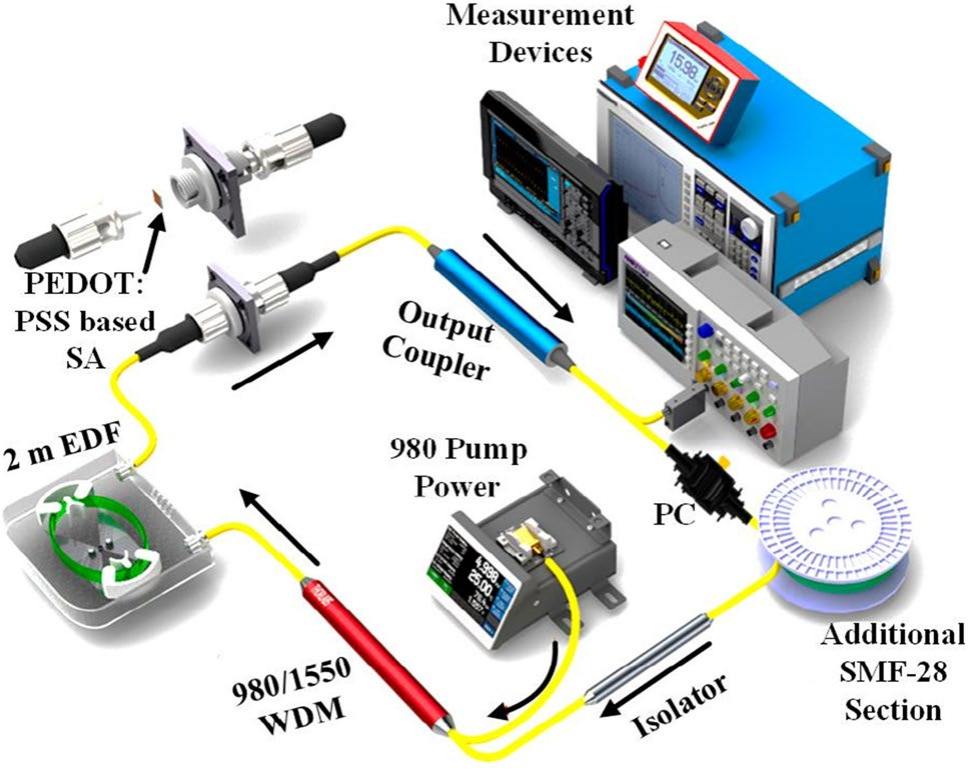
Laser configuration of the mode-locked EDFL operation.

In this section, mode-locked EDFL were demonstrated in 3 different cavity length and output coupler ratio. Figure 3 illustrates the proposed structure of the mode-locked laser, which contains a 0.5 m long WDM fiber, a 2.0 m long EDF, an optical isolator, an output coupler, a polarization controller (PC), and an additional single-mode fiber (SMF) section. The EDF has a core diameter, a numerical aperture, and an Erbium ion absorption of 4 μm, 0.16, and 23 dB/m at 980 nm. The 2 m erbium fiber allows electron lay on the lower occupancy of energy band during the population inversion process, and to induce lasing at longer wavelength. A 980 nm laser diode was utilized to pump the EDF through WDM. It generated photons that oscillated in the cavity to form a laser and tap out through the output coupler. An isolator was utilized to ensure that the unidirectional laser light was propagated into the ring cavity. A polarization controller (PC) was used to adjust the polarization state of oscillating laser to optimize the mode-locking process. The EDF, SMF, and WDM fiber has group velocity dispersion (GVD) of 27.6 ${\text{ps}}^{2}\text{/km}$, − 21.7 ${\text{ps}}^{2}\text{/km}$, and − 48.5 ${\text{ps}}^{2}\text{/km}$, respectively. A 50, 32, and 20 m long additional SMF were integrated into the ring laser cavity for three experiments. An oscilloscope (OSC) (INSTEK GDS-3352) and radio frequency (RF) spectrum analyzer (Anritsu MS2683A) were utilized to monitor the pulse train in time and frequency domain, respectively via an InGaAs fast photodetector. The optical spectrum analyzer (OSA) (YOKOGAWA AQ6370C) with a resolution of 0.02 nm to investigate the mode-locked laser in wavelength domain. While the optical power meter was used to gauge the output power of pulsed fiber laser operation. An autocorrelator (APE PulseCheck) was utilized to measure the pulse duration of the mode-locked pulses.

## Mode-locked EDFL performance

### Mode-locking performance at 60.7 m cavity length

At first, 50 m long SMF was added into the ring cavity while a 20:80 output coupler was utilized so that 20% of the output can be extracted for analysis. The 80% was looped back into the cavity to oscillate and interact with the SA for mode-locking pulse generation. In this experiment, the total cavity length was about 60.7 m, and the net cavity dispersion was estimated to be − 1.43 ${\text{ps}}^{2}$. The CW laser threshold at pump power of 10 mW. As LD power gradually increased to 134 mW, a self-started mode-locked was successfully generated. Despite operating at below the material bandgap, PEDOT: PSS: SA started mode-locked due to edge-related sub-bandgap cases^39^. The mode-locked EDFL was maintained up to a maximum pump power of 300 mW with a repetition rate of 3.417 MHz.

**Figure 4:**
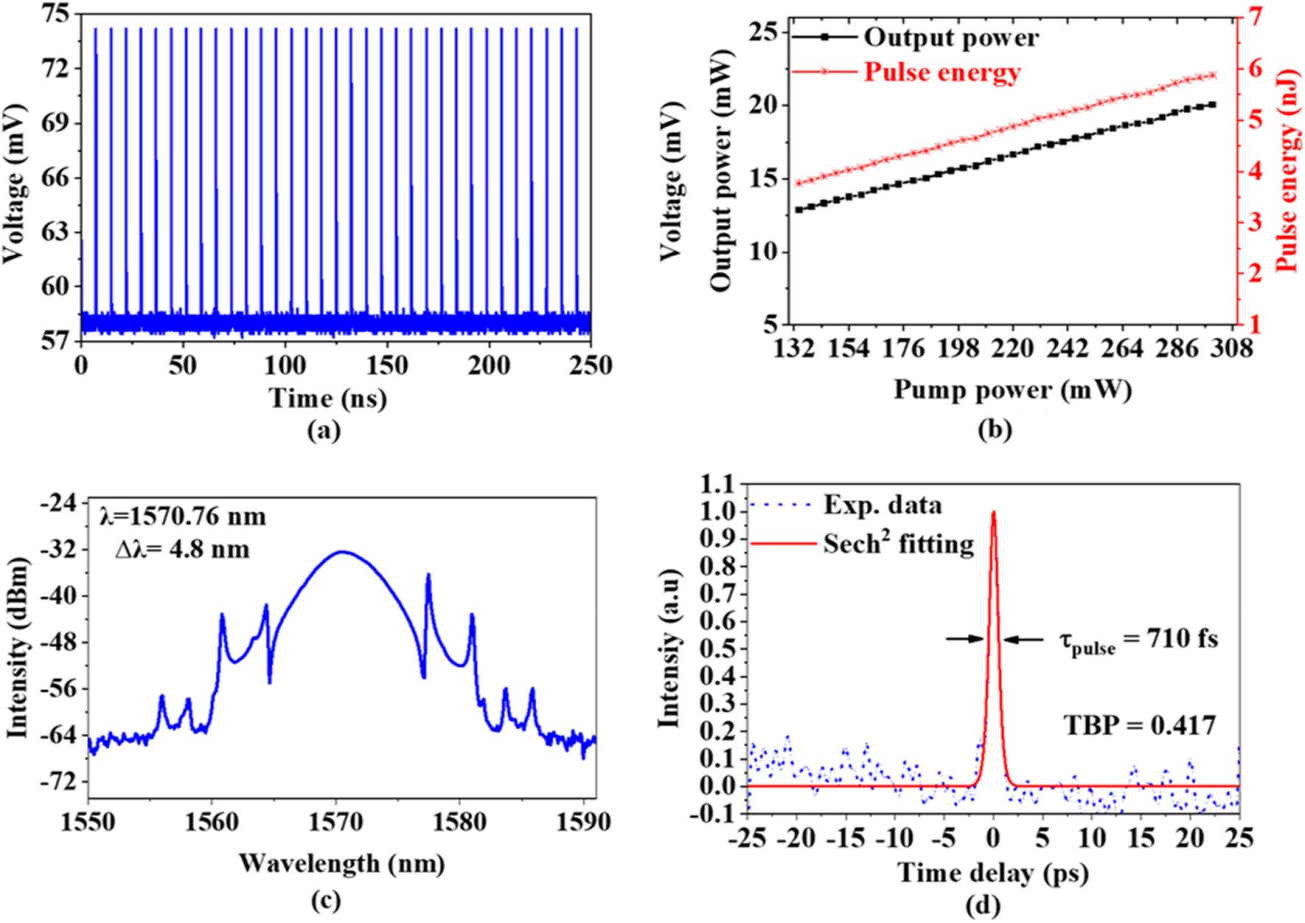
Characteristic of mode-locked EDFL operation (**a**) pulse train in time domain, (**b**) Pulse energy and output power changing with pump power (**c**) Optical spectrum, and (**d**) Autocorrelator trace.

The typical time domain characteristic of the mode-locked EDFL at maximum input LD power of 300 mW is shown in Fig. 4a. The pulse train was very regular, with a pulse repetition rate of 3.417 MHz and a pulse period of 292.6 ns, which corresponds to the cavity length in the erbium laser. The mode-locking was stably operating between the pump power range from 134 to 300 mW. Figure 4b illustrations the pulse energy and average output power versus input LD power. At pump power of 300 mW, the maximum pulse energy and average output power are achieved at 5.87 nJ and 20.07 mW, respectively.

The optical spectrum of the mode-locked EDFL at pump power of 300 mW is presented in Fig. 4c. The mode-locking pulse operated at a central wavelength of 1570.76 nm with 3-dB bandwidth of 4.8 nm. The Kelly's sidebands were observed in the soliton spectrum. This was predictable as mode-locked EDFL was operating in the anomalous dispersion regime which facilitated soliton pulse shaping through self-phase modulation (SPM) and the GVD interplay. The slight dips at the sideband are attributed to the effect of four-wave mixing (FWM) between the soliton and dispersive wave that induced by the periodic energy exchange in the fiber laser^40^. It corresponded to Kelly’s sidebands which are visible on both sides of the optical spectrum, as the total dispersion value of the cavity was measured to be around − 1.43 ${\text{ps}}^{2}$. The autocorrelation trace of the soliton mode-locking laser is presented in Fig. 4d. The pulse pattern follows on ${\text{sech}}^{2}$ pulse profile with a duration of 710 fs and a time-bandwidth product (TBP) was measured to be ~ 0.414, which indicates the pulse is slightly chirped. This chirp may be partially due to the third-order dispersion. Another factor may be spectral filtering through the non-uniform erbium gain medium^41^. It also observes that the change in room temperature does not give an impact on the mode-locked EDFL performance.

**Figure 5:**
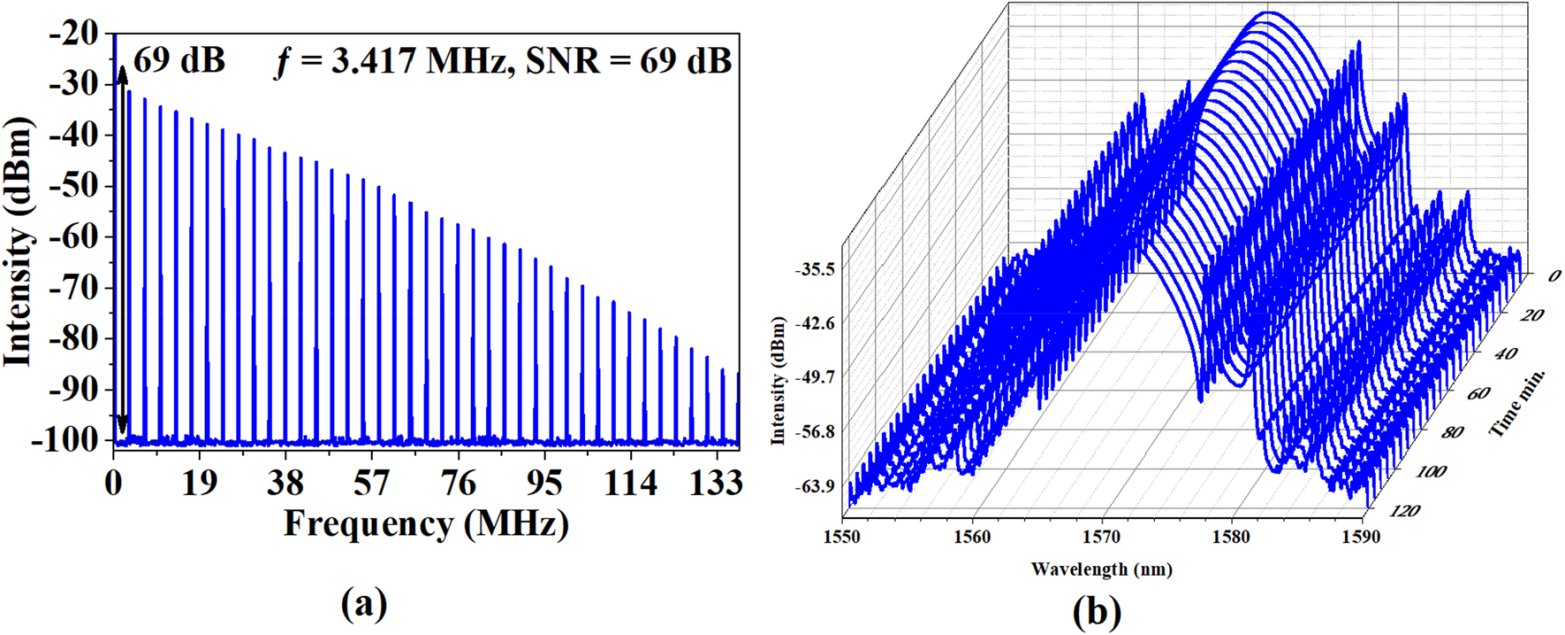
Mode-locked EDFL performance with 60.7 m cavity length (**a**) RF spectrum and (**b**) Long-term stability.

Figure 5a illustrations the RF spectrum of mode-locked EDFL operation at 300 mW input power and 138 MHz frequency span. The fundamental frequency was recorded to be 3.417 MHz which corresponds to laser cavity length, and it is estimated based on the equation $\text{f=c/nL}$, as c is the speed of the light, n is the refractive index of an optical fiber, and L is cavity length. As L is 60.7 m, c is 3 × 10^8^ and n is 1.44 at 1500 nm, the fundamental frequency was estimated at 3.432 MHz. The theoretical calculations of the frequency correspond to the frequency of the experimental work, which achieved about 3.417 MHz. The 69 dB SNR at 3.417 MHz proven the stability of mode-locking operating in the cavity^42^. The long-term evaluation of mode-locked EDFL operation is presented in Fig. 5b. The stable soliton mode-locked EDFL was generated in the laboratory for up to 2 h without any noticeable decay of pulse train and RF spectrum performance. The output spectra were taken each 5 min for a total period of 2 h, as the central wavelength, 3-dB bandwidth, and peak wavelength were steadily operated at 1570.76 nm, 4.8 nm, and − 32.34 dBm, respectively.

### Mode-locking performance at 42.7 m cavity length

**Figure 6:**
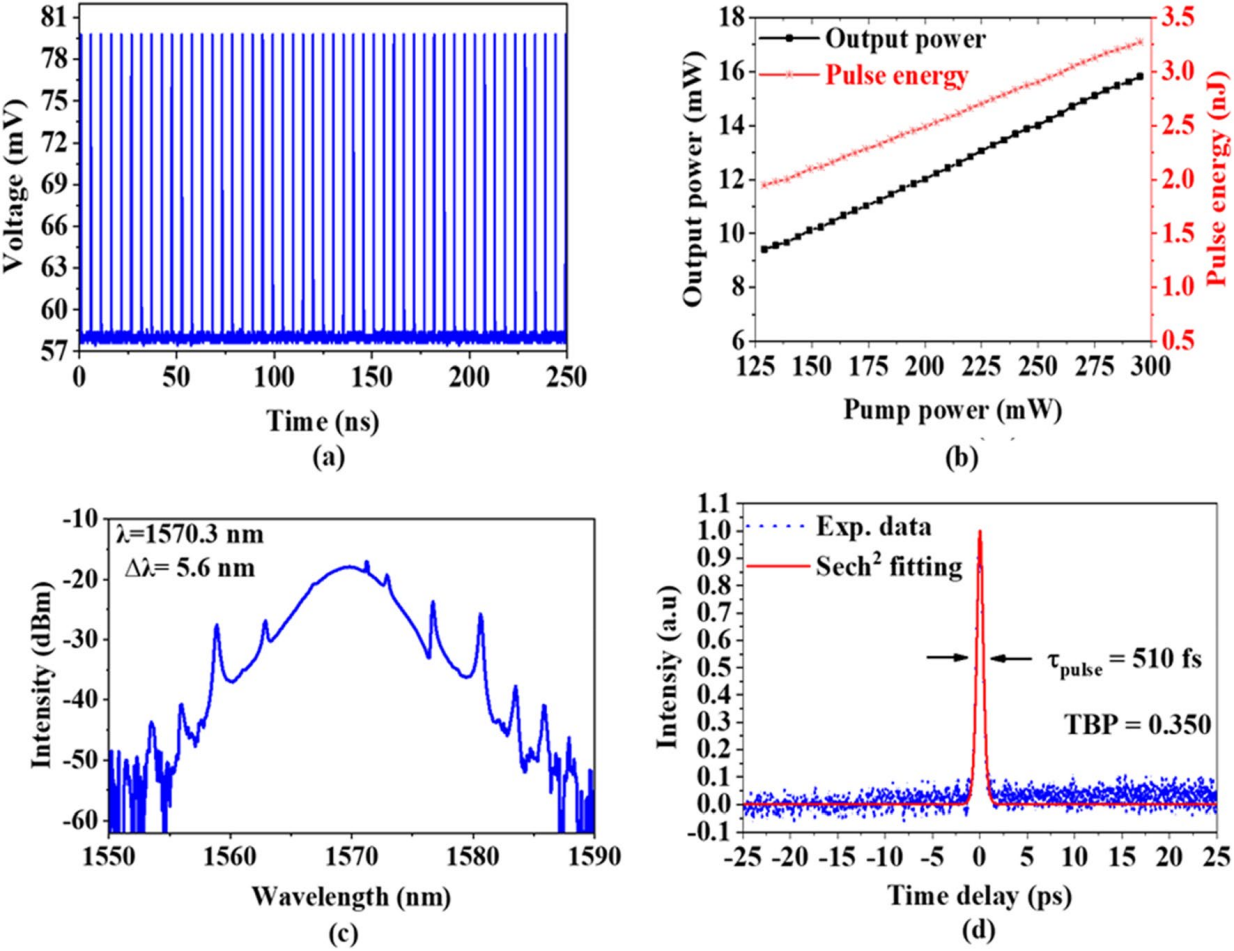
Temporal and spectral characteristics of the soliton pulse with 35 m cavity length (**a**) typical pulse train, (**b**) Pulse energy and output power changing with pump power (**c**) output spectrum, and (**d**) autocorrelator trace.

In the second experiment, 32 m long SMF was added into the EDFL cavity to form the cavity length of 42.7 m with the net cavity dispersion of – 1.02 ${\text{ps}}^{2}$. A 90:10 output coupler was used instead of an 80:20 coupler to obtain the optimum result. The mode-locked pulse was realized as a smaller threshold input LD power of 129 mW. The mode-locking operation was maintained up to a pump power of 295 mW. Figure 6a illustrations the mode-locked EDFL pulse train with a pulse repetition rate of 4.831 MHz at the maximum pump power of 295 mW. The pulse period was measured at about 207 ns, which matches well with the cavity length. The fundamental frequency was estimated according to the cavity length which was about 4.879 MHz based on the previous equation, as the total cavity length was about 41.7 m. Figure 6b illustrations the pulse energy and output power of the mode-locked EDFL operation as they are plotted versus input LD power. As the pump power was increased from 129 to 295 mW, the pulse energy increased from 1.94 to 3.27 nJ, whereas the output power increased from 9.41 to 15.82 mW, respectively.

The output spectrum of the mode-locked EDFL operation is presented in Fig. 6c. The mode-locked was operated at a central wavelength of 1570.3 nm with a 3-dB bandwidth of 5.6 nm. The laser operated at a shorter wavelength compared to that of the previous cavity due to the use of shorter cavity length and 90:10 coupler, which in turn reduces the total cavity loss. Figure 6d illustrations the autocorrelation tracing of the pulse duration measurement of mode-locked EDFL operation, as the pulse width was obtained of 510 fs. As predicted, the pulse width is shorter than the previous experiment due to the larger 3 dB bandwidth was recorded. The TBP was calculated to be ~ 0.35 which is closer to the transform-limited TBP for ${\text{sech}}^{2}$ pulses of 0.315 compared to the previous setup.

**Figure 7:**
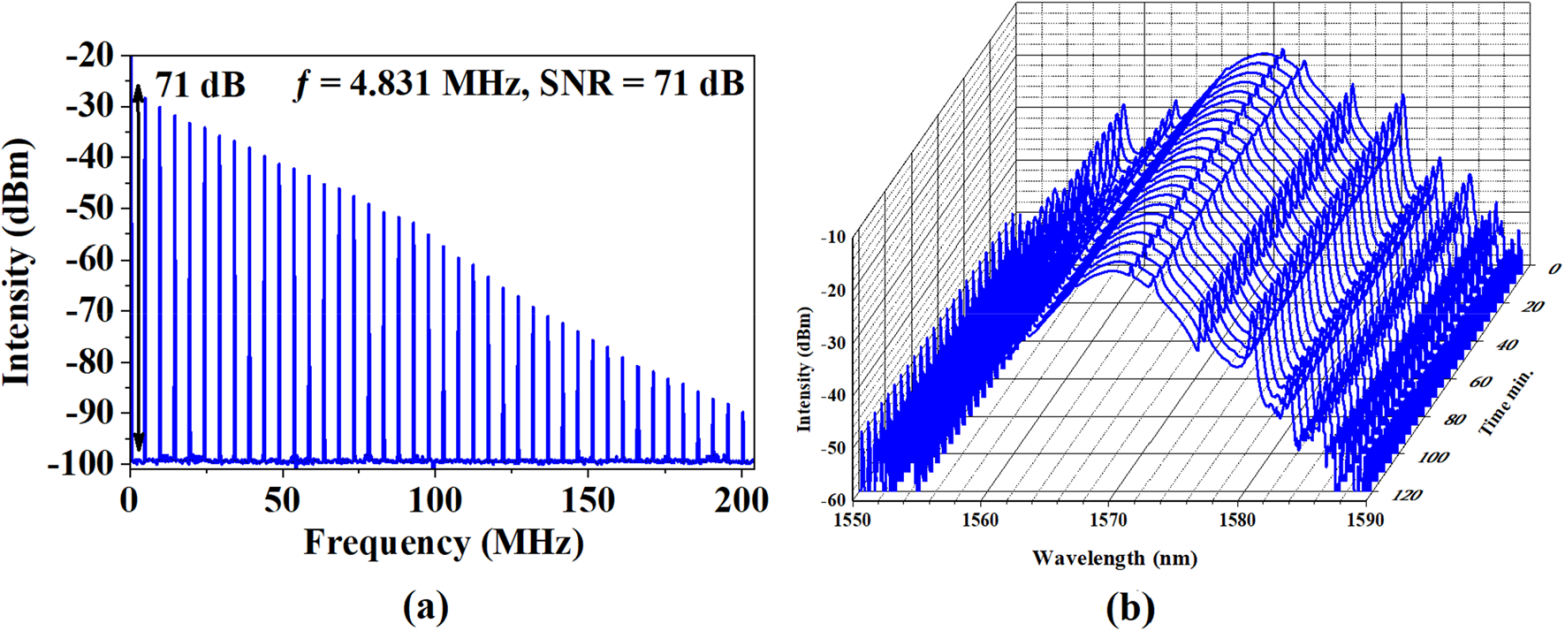
Mode-locked EDFL performance with 42.7 m cavity length (**a**) RF spectrum and (**b**) long-term stability.

Figure 7a illustrations the RF spectrum of the mode-locked operation. It shows a fundamental frequency at 4.831 MHz. with SNR of 71 dB. The stability of the laser operation was further investigated by conducting a 120 min observation as shown in Fig. 7b. The laser operation was operated stably without any noticeable decay of pulse train and RF spectrum performance.

### Mode-locking performance at 33.7 m cavity length

**Figure 8:**
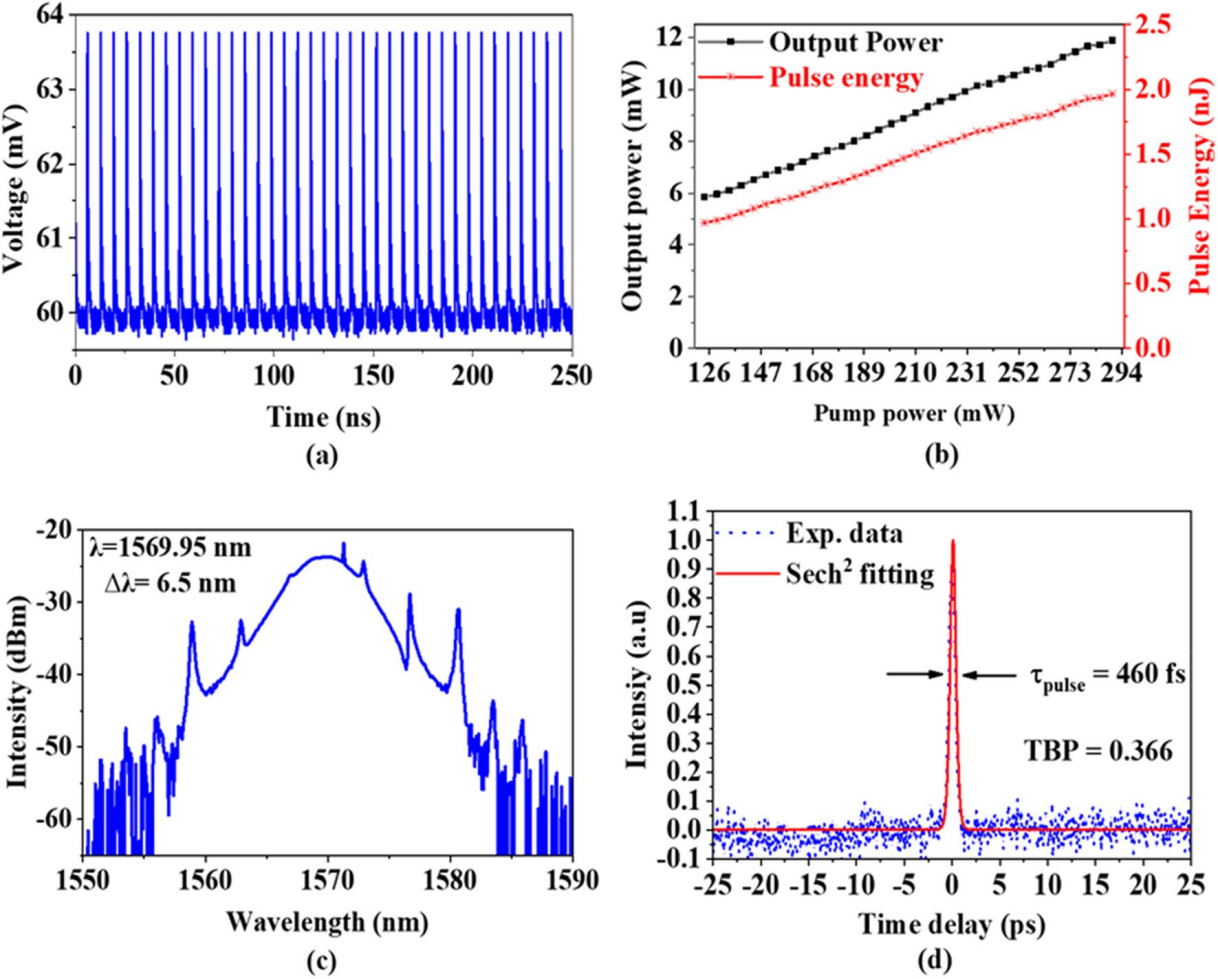
Spectral and temporal properties of the soliton pulse with 33.7 m cavity length (**a**) typical pulse train, (**b**) Pulse energy and output power changing with pump power (**c**) laser wavelength (**d**) autocorrelator trace.

In the third experiment, the cavity length is further reduced to 33.7 m to improve the mode-locking performance of the laser. A 20 m long additional SMF was integrated into the laser ring cavity to achieve the cavity dispersion of − 0.84 ${\text{ps}}^{2}$. A 95:5 output coupler was used in the proposed cavity to further reduce loss in the laser cavity to obtain a shorter pulse width. A self-started soliton pulse was successfully produced at the threshold pump power of 124 mW. The laser operated at a constant pulse repetition rate of 6.049 MHz between pump power of 124 to 290 mW. Figure 8a illustrations a typical pulse train at pump power of 290 mW, indicating an identical mode-locked pulse train with no significant instabilities nor distortion. The pulse period is about 165.3 ns, which corresponds to the cavity length and repetition rate. The pulse energy and average output power were increased linearly with the increase of pump power, as recorded in Fig. 8b. At the maximum pump power of 290 mW, the pulse energy and average output power are measured to be 1.96 nJ and 11.89 mW, respectively.

Figure 8c presents the output optical spectrum at the pump power of 290 mW. The laser operated as soliton spectrum at a central wavelength of 1569.95 nm with a 3 dB bandwidth of 6.5 nm. The mode-locked EDFL operated at anomalous cavity dispersion and proved by Kelly sidebands^43^. The laser wavelength demonstrated symmetrical Kelly sidebands with a distance to the central wavelengths of 2.9, 6.9, and 10.9 nm for the first, second, and third^-^order, respectively. The distance is related to the pulse duration, operating wavelength, and total net dispersion^44^. Figure 8d shows the autocorrelation trace with sech^2^ fitting which has a pulse width of 460 fs. The TBP is 0.363 which is closing to the transform-limited value of 0.315, indicating the pulse is slightly chirped.

**Figure 9:**
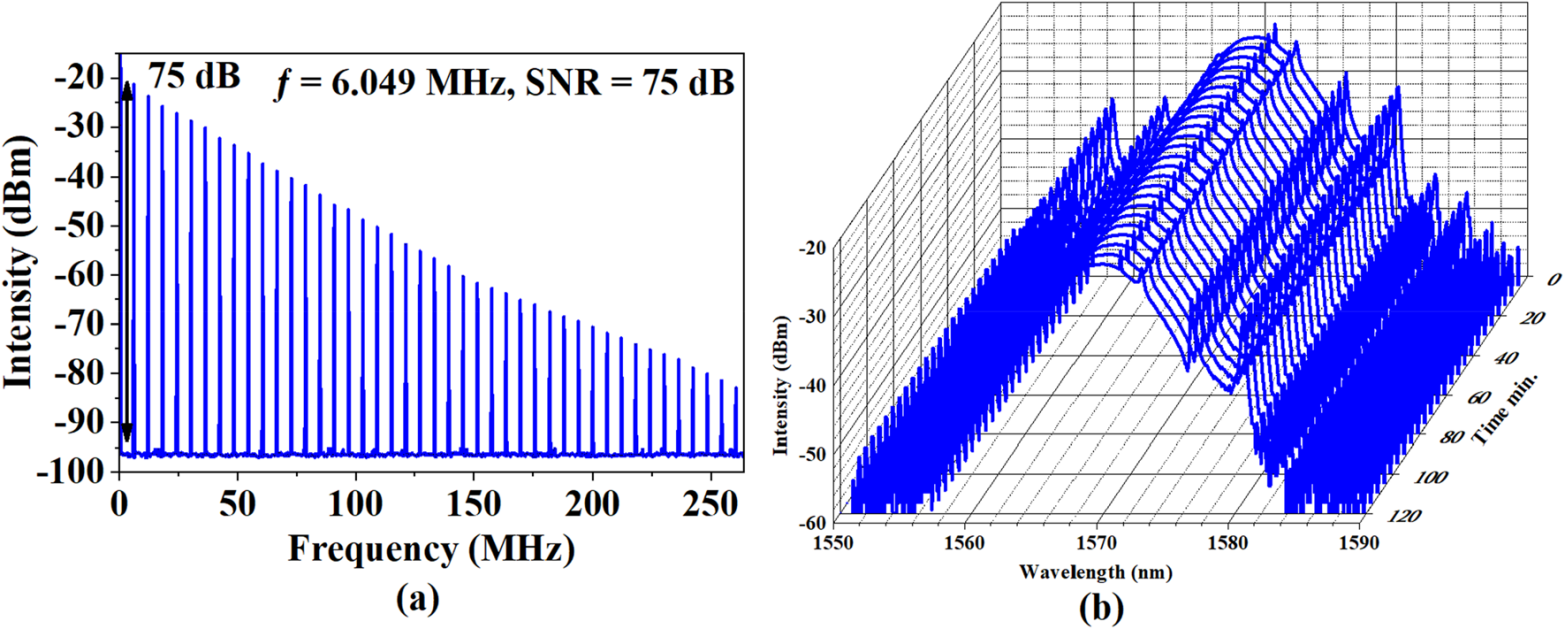
Mode-locked EDFL performance with 33.7 m cavity length (**a**) RF spectrum and (**b**) long-time evaluation.

The RF output spectrum was recorded as shown in Fig. 9a. The fundamental frequency of the laser (6.049 MHz) has a very good signal-to-noise ratio (SNR) of ~ 75 dB, which further verified the stability of the laser. The long-term evaluation of laser operation is presented in Fig. 9b. The mode-locked EDFL operated stably in the laboratory for up to 2 h without any noticeable decay of pulse train and RF spectrum performance. The output spectra were taken each 5 min for a total period of 2 h, as the central wavelength, 3-dB bandwidth, and peak intensity of spectrum were kept at 1569.95 nm, 6.5 nm, and − 23.69 dBm, respectively.

All experiments generated stable pulse trains with excellent performance for the development of high-power mode-locked lasers. Table 1 shows the three mode-locked EDFL operations based on cavity length and output coupler ratio. The three different mode-locked have achieved a high stability output laser with excellent performance. From the experiments, PEDOT: PSS has proven as exceptional SA function to induce ultrafast laser with good long-term stability and reusability in different dispersion cavity.

**Table 1:** Summary of performance of three mode-locked EDFL operations.

Experiments	Cavity length (m)	Type of OC (%)	Central wavelength (nm)	Repetition rate (MHz)	Pulse width (fs)	Max. Pulse energy (nJ)	Max. Output power (mW)	SNR
First	60.7	80:20	1570.76	3.417	710	5.87	20.07	69 dB
Second	42.7	90:10	1570.3	4.831	510	3.27	15.82	71 dB
Third	33.7	95:5	1569.95	6.049	460	1.96	11.89	75 dB

Along the development of saturable absorber, many materials have been reported to induce mode-locking operation in fiber laser system, including TMDs^45–50^, TIs^51–53^, MXene^54^ and other emerging materials^55–58^. These materials have proven the capability to induce ultrafast phenomena, ranging from picosecond down to femtosecond. As compared to this work, we have achieved pulse width of 460 fs with pulse energy of 1.96 nJ. Even though this is not the best result among the reported work, but it is comparable to most of the reported literatures. More importantly, PEDOT: PSS as family member of OM, it inherited exceptional physical characteristics, including thermal stability and film-forming capability. The reported results in this work proven the great potential of PEDOT: PSS as a saturable absorber. Thus, it could be an alternative as saturable absorber material to cater the difference needs in the industry.

## Conclusion

Ultrafast lasers were successfully demonstrated using PEDOT: PSS-based SA into ring cavities operated in a L-band. The first SA was obtained by embedding the PEDOT: PSS into PVA film. It has a modulation depth of 50% with a saturation intensity of 0.15 ${\text{M}}{\text{W/cm}}^{2}$. Three different soliton mode-locked pulses were demonstrated based on three different cavity lengths. The laser operated at the pulse repetition rate/width of 3.417 MHz/710 fs, 4.831 MHz/510 fs, and 6.049 MHz/460 fs by utilizing an optical coupler (OC)/ cavity length of 20:80/60.7, 10:90/42.7, and 5:95/33.7 m, respectively. These lasers produced stable soliton pulses operating at a central wavelength of around 1570 nm.

## Supplementary Information

Supplementary Information 1.

Supplementary Information 2.

Supplementary Information 3.

Supplementary Information 4.

Supplementary Information 5.

Supplementary Information 6.

Supplementary Information 7.

Supplementary Information 8.

Supplementary Information 9.

Supplementary Information 10.

Supplementary Information 11.

Supplementary Information 12.

Supplementary Information 13.

Supplementary Information 14.

Supplementary Information 15.

## Data Availability

All data generated or analyzed during this study are included in this published article [and its supplementary information files].
